# Sex-specific adverse neonatal outcomes and umbilical cord arterial ph at birth: a population-based cohort study

**DOI:** 10.1186/s12887-026-07865-5

**Published:** 2026-10-27

**Authors:** Wazhma Nassery Akel, Sara Abdulilah, Mehreen Zaigham

**Affiliations:** 1 Department of Obstetrics and Gynaecology, Skåne University Hospital, Malmö, 205 01 Sweden; 2 Obstetrics and Gynaecology, Institution of Clinical Sciences Lund, Lund University, Lund, Sweden

**Keywords:** Male, Neonate, Outcomes, Sex, Umbilical cord pH

## Abstract

**Background:**

Biological sex may influence neonatal vulnerability to adverse perinatal outcomes, but sex-related differences in these outcomes remain underexplored. Male neonates may be more susceptible to perinatal asphyxia, metabolic acidosis, preterm birth, and morbidity, possibly due to sex-specific placental differences and inflammatory responses. Umbilical cord arterial pH (UApH) < 7.05 is used in Sweden to define neonatal acidemia, associated with poor outcomes. This study aimed to investigate the association between neonatal sex and adverse outcomes, focusing on UApH.

**Methods:**

This retrospective multicenter cohort study used data from the Swedish Perinatal Revision South Register (1995–2015). The cohort included live-born, singleton, vaginal births. Neonatal and maternal data were extracted, and logistic regression models compared outcomes between male and female neonates, adjusting for maternal age, gestational age, and birth weight.

**Results:**

Among 87 840 neonates, 51.6% were male. Male neonates had significantly higher adjusted odds of adverse outcomes compared with females: UApH < 7.05 (OR 1.18, 95% CI 1.07–1.30; *P* < 0.001), Apgar score < 7 at 5 min (OR 1.33, 95% CI 1.14–1.56; *P* < 0.001), CPAP use (OR 1.66, 95% CI 1.46–1.87; *P* < 0.001), NICU admission (OR 1.51, 95% CI 1.43–1.60; *P* < 0.001), hypoxic-ischemic encephalopathy (OR 2.24, 95% CI 1.29–3.90; *P* = 0.002) and central nervous system disease (OR 1.52, 95% CI 1.30–1.76; *P* < 0.001).

**Conclusions:**

Male neonatal sex was associated with several adverse perinatal outcomes, including acidemia and neurological complications. These findings contribute to the understanding of sex-related differences in neonatal vulnerability and warrant further investigation.

**Trial registration:**

Not applicable.

## Introduction

Biological sex may influence neonatal vulnerability to adverse perinatal outcomes, but sex-related differences in these outcomes remain underexplored. Previous studies have suggested that male neonates may be more susceptible to perinatal asphyxia, metabolic acidosis and death [1–3]. Other studies have found an increased risk of spontaneous preterm birth among male neonates, including those with intact membranes and those with preterm premature rupture of membranes [2], others have found no significant differences in high-risk populations based on prior obstetric history [4]. In contrast, among low-risk pregnancies, male sex has been associated with higher preterm birth rates [5]. Emerging data also suggest that sex-based placental differences may contribute to this risk. In particular, male placentas may exhibit a higher prevalence of chronic inflammatory lesions in very preterm births (< 32 weeks’ gestation) [6].

It is well-known that umbilical cord blood gas analysis, particularly umbilical cord arterial pH (UApH), provides critical information about the neonate’s metabolic condition at birth and serves as an early marker of potential complications [7–9]. In Sweden, neonatal acidemia is defined as a UApH < 7.05 [10]. Studies have consistently shown associations between neonatal acidemia and adverse clinical outcomes, including low Apgar scores, admission to neonatal intensive care unit (NICU), and the need for respiratory resuscitation [11–13]. The use of UApH for the timely identification of acidosis is essential for reducing the risk of mortality and neurological sequelae [14].

Although previous studies have reported an association between male neonatal sex and adverse perinatal outcomes, few large population-based studies have simultaneously evaluated the association between neonatal sex, umbilical artery pH, and multiple clinically relevant neonatal outcomes within the same cohort using validated umbilical cord blood gas measurements.

Therefore, the aim of this study was to investigate whether male neonatal sex is independently associated with umbilical artery pH < 7.05 and other adverse neonatal outcomes in a large population-based cohort from southern Sweden.

## Methods

### Study design and setting

A retrospective, multicenter, population-based cohort study was conducted using data collected from seven maternity units in the South of Sweden. Data was extracted from the Perinatal Revision South Register from the year 1995 to 2015. The study period was determined by the availability of data from the Perinatal Revision South Register, which was closed in 2015. Follow-up in the register was limited to the neonatal period, defined as the first 28 completed days after birth (day 0–27), with no subsequent post-neonatal follow-up. The Perinatal Revision South Register was a regional clinical quality register incorporating routinely collected obstetric and neonatal data from seven maternity units in southern Sweden. The dataset available for the present study did not contain metadata specifying which individual healthcare professional entered each variable, the precise timing of individual data entries, or measures of inter-center consistency. This study followed the Strengthening the Reporting Observational Studies in Epidemiology (STROBE) guidelines for reporting observational studies [15].

### Study participants

The dataset consisted of 104 072 singleton, live births. Umbilical arterial and venous cord blood samples were collected immediately after birth using pre-heparinized syringes and analyzed within 10–15 min using a blood gas analyzer located in the maternity unit. UApH samples had already been validated prior to the execution of the study. Briefly, cases with missing data on either umbilical venous pH (UVpH) or umbilical artery pH (UApH) were excluded. Samples with a veno-arterial difference of ≤ 0.02 were excluded to ensure inclusion of samples taken from different vessels. Additionally, outliers with an UVpH of more than 7.50 or an UApH of less than 6.50 were removed [16].

Both male and female neonates were included, and neonatal sex was determined at birth by visual examination. All cases with missing data on neonatal sex and the neonatal sex unknown/uncertain at birth were removed from the study population. Maternal age, parity, history of previous cesarean section (CS), mode of birth, Body Mass Index (BMI), maternal smoking, and how labor began (birth start) were reported in the dataset. No information regarding race or ethnicity was collected in the register.

### Outcome variables

The primary outcome was UApH < 7.05, used as a proxy for neonatal acidemia. Secondary outcomes included Apgar score < 7 at 5 min, use of CPAP, admission to the NICU, development of seizures, HIE I–III, and central nervous system (CNS) disease. CNS disease was a predefined registry variable indicating suspected or confirmed pathology involving the central nervous system (brain or spinal cord) during the neonatal period. The registry variable did not distinguish between specific CNS diagnoses. Each of these outcomes was categorized dichotomously, as ‘yes’ (indicating the occurrence of the event), and ‘no’ (indicating the absence of the event). UApH was divided into < 7.05 and ≥ 7.05, with UApH ≥ 7.05 considered normal. Apgar score at 5 min was categorized as ‘<7 points’ (abnormal) and ‘≥7 points’ (normal).

### Statistical methods

Chi^2^-tests were used to assess differences in baseline characteristics between sexes. The influence of neonatal sex was correlated to umbilical cord arterial pH and adverse perinatal outcomes using logistic regression, calculating Odds ratios (OR), Confidence intervals (CI), as well as p-values. First, this was done without the adjustment for covariates, specifically the univariate analysis. Consequently, adjustments for gestational age, maternal age, and birth weight were included in the model, creating a multivariate analysis to better assess the relation between neonatal sex and adverse perinatal outcomes while accounting for confounding variables. Missing data was addressed as ‘missing’ in each variable. A p-value < 0.05 was considered statistically significant in the primary analyses. To account for multiple comparisons across the seven outcomes, a sensitivity analysis was performed using a Bonferroni-corrected significance threshold of 0.05/7 = 0.0071. Statistical analysis was performed using IBM SPSS version 29.0 (Armonk, NY: IBM Corp).

## Results

**Fig. 1:**
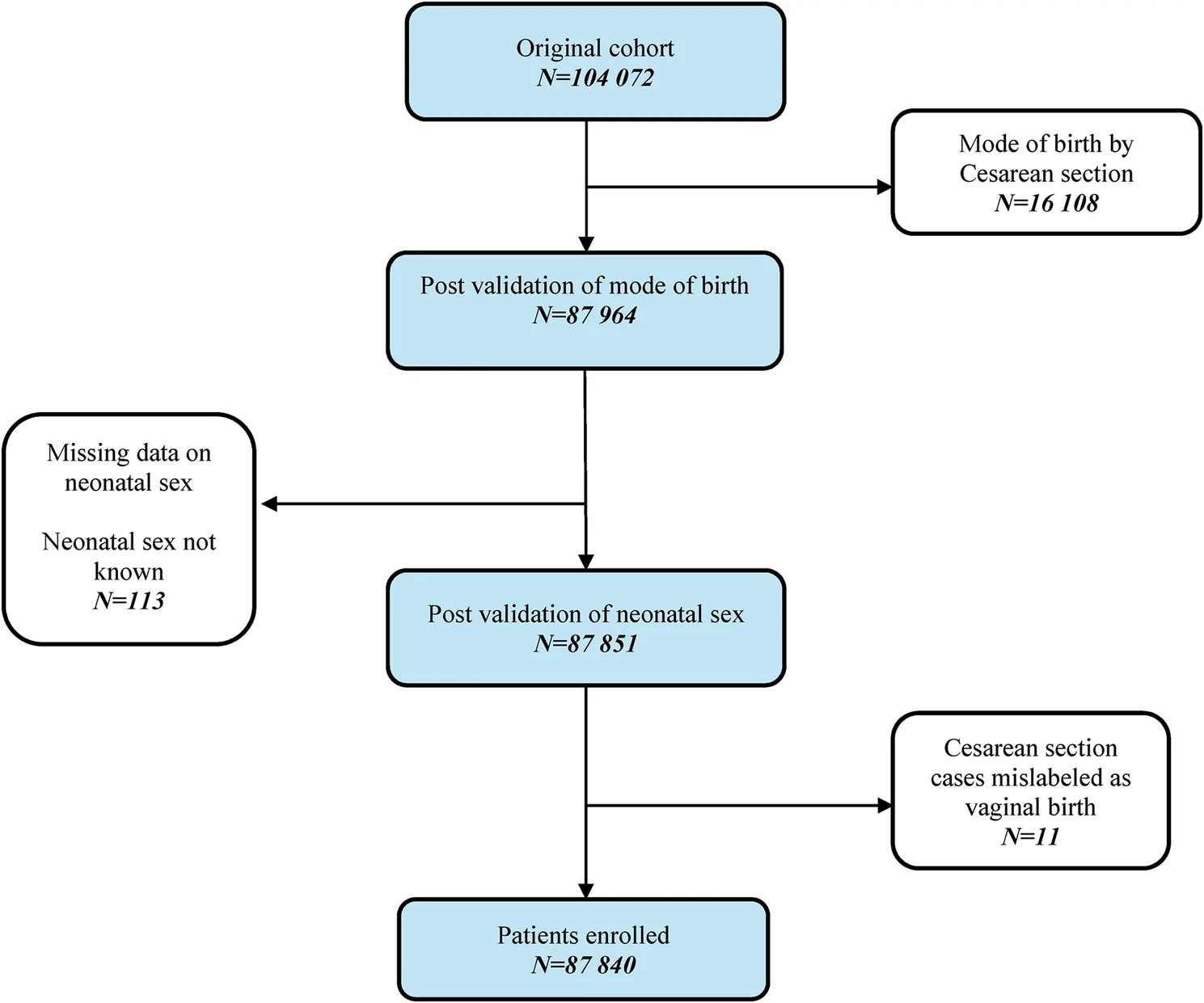
Flow chart of study population

Of the initial 104 072 births, neonates delivered by cesarean section were excluded, yielding 87 964 cases. An additional 113 cases were excluded due to missing or indeterminate neonatal sex. During data validation, 11 cases were identified as incorrectly labelled ‘vaginal births’ (based on ‘birth start’ data) and were removed. The final study population comprised 87 840 neonates **(**Fig. 1**)**.

Most mothers were 20–34 years of age (70 298/87 840, 80.0%) (*P* = 0.112). Both nulliparous and multiparous women were well represented (86 874/87 840, 98.9%) (*P* = 0.335). Regarding birth mode, a higher percentage of male neonates were born vaginally with the use of an instrument (3938/45 356, 8.7%) such as forceps or vacuum extraction, compared to female neonates (2977/42 484, 7.0%) (*P* < 0.001). Labor induction was also more frequent in males (4363/45 356; 9.6%) than in females (3697/42 484, 8.7%; *P* < 0.001) (Table 1). An increased percentage of male neonates had an Apgar score of 4–6 (311/45 356, 0.7%), in comparison to females (228/42 484, 0.5%) (*P* = 0.017). Similarly, for Apgar scores in the range of 0–3, more male neonates (60/45 356, 0.1%) had a low Apgar score at the age of 5 min in comparison to females (45/42 484, 0.1%) (*P* = 0.017) (Table 1).

**Table 1:** Baseline characteristics of the study population stratified by neonatal sex

Baseline Characteristics	Total		Male		Female		Chi2-test
	*N*	%	*N*	%	*N*	%	*P*-value
**Total**	87 840	100	45 356	100	42 484	100	
**Maternal age**,** years**							0.112
< 20	1813	2.1	965	2.1	848	2.0	
20–34	70 298	80.0	36 374	80.2	33 924	79.9	
35–39	13 379	15.2	6842	15.1	6537	15.4	
≥ 40	2350	2.7	1175	2.6	1175	2.8	
							0.335
**Parity**							
Nulliparous	40 025	45.6	20 565	45.3	19 460	45.8	
Multiparous	46 849	53.3	24 282	53.5	22 567	53.1	
Missing	966	1.1	509	1.1	457	1.1	
**Previous Cesarean section**							0.319
Yes	2857	3.3	1449	3.2	1408	3.3	
No	84 983	96.7	43 907	96.8	41 076	96.7	
**Birth mode**							**< 0.001**
Vaginal, non-instrumental	80 925	92.1	41 418	91.3	39 507	93.0	
Vaginal, instrumental	6915	7.9	3938	8.7	2977	7.0	
**BMI** ^**a**^							0.217
< 18.5	1260	1.4	618	1.4	642	1.5	
18.5–24.99	30 792	35.1	15 853	35.0	14 939	35.2	
25–29.9	13 537	15.4	6951	15.3	6586	15.5	
≥ 30	6486	7.4	3389	7.5	3097	7.3	
Missing	35 765	40.7	18 545	40.9	17 220	40.5	
**Maternal smoking**							0.497
No	56 896	64.8	29 342	64.7	27 554	64.9	
Yes, 1–9/day	3750	4.3	1959	4.3	1791	4.2	
Yes, ≥ 10/day	1400	1.6	706	1.6	694	1.6	
Missing	25 794	29.4	13 349	29.4	12 445	29.3	
**Birth start**							**< 0.001**
Spontaneous	79 773	90.8	40 990	90.4	38 783	91.3	
Induction	8060	9.2	4363	9.6	3697	8.7	
Missing	7	0.0	3	0.0	4	0.0	
**Gestational duration in weeks**							**< 0.001**
< 32 + 0	198	0.2	117	0.3	81	0.2	
32 + 0–36 + 6	3403	3.9	1896	4.2	1507	3.5	
37 + 0–40 + 6	65 367	74.4	32 895	72.5	32 472	76.4	
41 + 0–41 + 6	14 439	16.4	7851	17.3	6588	15.5	
≥ 42 + 0	4388	5.0	2577	5.7	1811	4.3	
Missing	45	0.1	20	0.0	25	0.1	
**Birth weight in gram**							**< 0.001**
< 2500	1592	1.8	787	1.7	805	1.9	
2500–3999	69 193	78.8	34 075	75.1	35 118	82.7	
4000–4499	13 826	15.7	8351	18.4	5475	12.9	
≥ 4500	3195	3.6	2118	4.7	1077	2.5	
Missing	34	0.0	25	0.1	9	0.0	
**Apgar score at 5 min**							**0.017**
0–3	105	0.1	60	0.1	45	0.1	
4–6	539	0.6	311	0.7	228	0.5	
7–10	87 182	99.3	44 976	99.2	42 206	99.3	
Missing	14	0.0	9	0.0	5	0.0	

Abbreviations: ^a^Body Mass Index

**Fig. 2:**
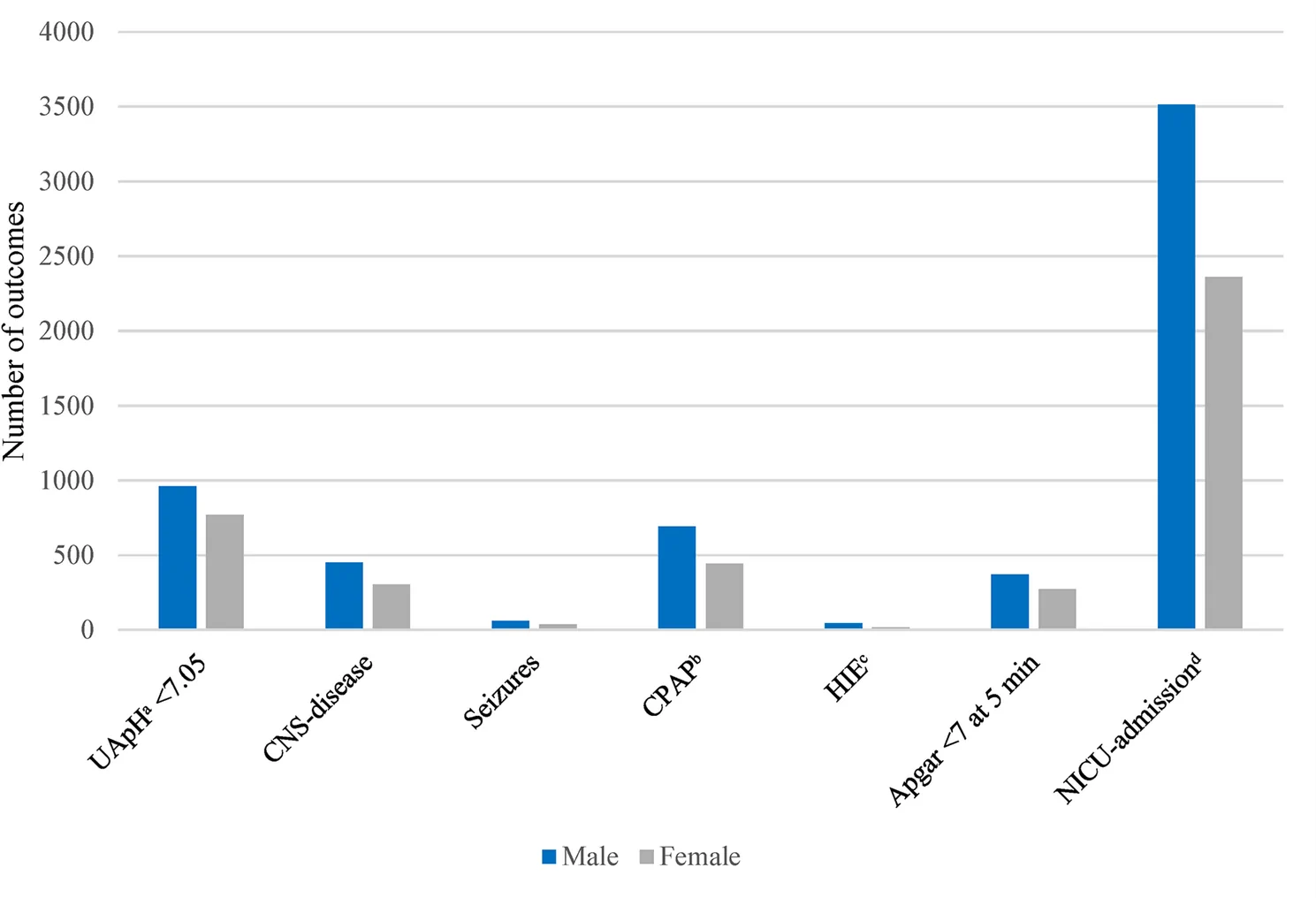
Number of adverse perinatal outcomes among male and female neonates in the study population. Abbreviations: aUmbilical artery cord pH. bContinuous Positive Airway Pressure. cHypoxic-Ischemic Encephalopathy. dNeonatal Intensive Care Unit

Adverse perinatal outcomes were more common among male neonates than females in the study population (Fig. 2). This pattern persisted across all adverse outcomes: UApH < 7.05 was more prevalent among male neonates (962/45 356, 2.1%) than females (768/42 484, 1.8%) (*P* = 0.001); similarly, Apgar < 7 occurred more frequently in males (371/45 356, 0.8%) compared to females (273/42 484, 0.6%) (*P* = 0.002). CPAP usage was higher among males (691/45 356, 1.5%) than females (442/42 484, 1.0%) (*P* < 0.001), and admission to the NICU was also more common in males (3516/45 356, 7.8%) compared to females (2361/42 484, 5.6%) (*P* < 0.001). Seizures occurred more often in males (62/45 356, 0.1%) than females (39/42 484, 0.1%) (*P* = 0.051), and males were more frequently diagnosed with HIE (45/45 356, 0.1%) compared to females (18/42 484, 0.04%) (*P* = 0.002). Finally, CNS disease was more prevalent in male neonates (451/45 356, 1.0%) than in females (304/42 484, 0.7%) (*P* < 0.001) (Table 2).

**Table 2:** Adverse perinatal outcomes in the study population stratified by neonatal sex

Perinatal outcome	Total		Male		Female		Chi2-test
	*N*	%	*N*	%	*N*	%	*P*-value
**Total**	87 840	100	45 356	100	42 484	100	
**UApH** ^**a**^							**0.001**
< 7.05	1730	2.0	962	2.1	768	1.8	
≥ 7.05	86 110	98.0	44 394	97.9	41 716	98.2	
**CNS-disease**							**< 0.001**
Yes	755	0.9	451	1.0	304	0.7	
No	87 085	99.1	44 905	99.0	42 180	99.3	
**Seizures**							0.051
Yes	101	0.1	62	0.1	39	0.1	
No	87 739	99.9	45 294	99.9	42 445	99.96	
**CPAP** ^**b**^							**< 0.001**
Yes	1133	1.3	691	1.5	442	1.0	
No	86 707	98.7	44 665	98.5	42 042	99.0	
**HIE** ^**c**^							**0.002**
Yes	63	0.1	45	0.1	18	0.04	
No	87 777	99.9	45 311	99.9	42 466	99.96	
**Apgar at 5 min**							**0.002**
< 7	644	0.7	371	0.8	273	0.6	
≥ 7	87 196	99.3	44 985	99.2	42 211	99.4	
**NICU-admission** ^**d**^							**< 0.001**
Yes	5877	6.7	3516	7.8	2361	5.6	
No	81 963	93.3	41 840	92.2	40 123	94.4	

Abbreviations: ^a^Umbilical artery cord pH. ^b^Continuous Positive Airway Pressure. ^c^Hypoxic-Ischemic Encephalopathy ^d^Neonatal Intensive Care Unit. Assumption that missing values did not have the adverse outcome

**Table 3:** Logistic regression representing Odds ratios (OR) and confidence intervals (CIs) comparing male to female neonatal sex with perinatal outcomes

Perinatal outcome	Unadjusted			Adjusted*		
	OR	95% CI	*P*-value	OR	95% CI	*P*-value
UApH < 7.05^a^	1.18	1.07–1.30	**0.0008**	1.18	1.07–1.30	**< 0.001**
CNS-disease	1.39	1.20–1.61	**< 0.001**	1.52	1.30–1.76	**< 0.001**
Seizures	1.49	0.99–2.22	0.051	1.55	1.03–2.33	**0.009**
CPAP^b^	1.47	1.30–1.66	**< 0.001**	1.66	1.46–1.87	**< 0.001**
HIE^c^	2.34	1.36–4.05	**0.001**	2.24	1.29–3.90	**0.002**
Apgar < 7 at 5 min	1.28	1.09–1.49	**0.002**	1.33	1.14–1.56	**< 0.001**
NICU-admission^d^	1.43	1.35–1.51	**< 0.001**	1.51	1.43–1.60	**< 0.001**

*Adjusted for maternal age, gestational age, and birth weight. ^a^Umbilical artery cord pH. ^b^Continuous Positive Airway Pressure

^c^Hypoxic-Ischemic Encephalopathy. ^d^Neonatal Intensive Care Unit

Using logistic regression, neonatal sex was compared to adverse perinatal outcomes (Table 3). Male neonatal sex was compared to female neonatal sex. After adjusting for maternal age, gestational age, and birth weight, male neonates had higher odds of UApH < 7.05 [OR 1.18; 95% CI: 1.07–1.30, *P* < 0.001], Apgar scores < 7 at 5 min [OR 1.33; 95% CI: 1.14–1.56, *P* < 0.001], CPAP [OR 1.66; 95% CI: 1.46–1.87, *P* < 0.001], and NICU-admission [OR 1.51; 95% CI: 1.43–1.60, *P* < 0.001]. Moreover, in the multivariate analysis, male neonates had a higher OR of developing seizures as well [OR 1.55; 95% CI: 1.03–2.33, *P* = 0.009]. In the sensitivity analysis using the Bonferroni-corrected significance threshold of *P* < 0.0071, the associations with UApH < 7.05, Apgar score < 7 at 5 min, CPAP use, NICU admission, HIE, and CNS disease remained statistically significant, whereas the association with seizures did not.

## Discussion

In this large, population-based cohort study, male neonatal sex was associated with several adverse perinatal outcomes, specifically UApH < 7.05, Apgar score < 7 at 5 min, the need for CPAP, admission to the NICU, HIE, and CNS disease.

A national cohort study from the Netherlands by Peelen et al. [2] analyzed the effect of sex on composite neonatal morbidity by gestational week in preterm infants, where they included outcomes such as sepsis, meconium aspiration syndrome, and necrotizing enterocolitis. The study concluded that the male sex has a significantly greater relative risk (RR) of developing these outcomes, not only in preterm but also in term neonates born ≥ 37 + 0 weeks of gestation. Moreover, Aibar et al. [3] also investigated the consequence of sex on perinatal outcomes in a retrospective cohort study where they demonstrated that UApH < 7.05 was more commonly observed in male neonates than female neonates, and admitted to the NICU and had an Apgar < 7 at 5 min of birth. However, solely UApH was a significant value after adjusting for confounders [3]. Further, a literature review by Di Renzo et al. [17] discussed the consequence of sex at birth and perinatal events, summarizing that more male than female neonates experienced cord abnormalities, acute fetal distress, UApH ≤ 7.05, along with intrauterine fetal death. In conjunction with this, a population-based study by Sheiner et al. [18] identified male sex as a risk factor in adverse perinatal outcomes, specifically in low Apgar scores at 5 min. These publications support the findings of the current study.

Although several associations were statistically significant, the absolute differences were small for some outcomes. For example, UApH < 7.05 occurred in 2.1% of male compared with 1.8% of female neonates, corresponding to an absolute difference of approximately 0.3% points. This should be considered when interpreting the clinical relevance of the observed associations.

It remains unclear what the underlying mechanism prompting males to be more prone to adverse outcomes in the first few weeks of life. As mentioned earlier, a study by Ghidini et al. [6] describes the differences in placentas according to fetal sex. The study found neonatal differences in placental histology, with a significantly greater number of inflammatory lesions at the implantation site in placentas carrying a male offspring. The authors suggested that this might be an immune response from maternal tissue against the invading interstitial trophoblasts of the male fetus [6]. Moreover, in a review by Clifton [19], it is proposed that during negative intrauterine environments, such as chronic maternal asthma, male neonates continue to grow despite these conditions, putting them at risk during stressful events such as acute asthma exacerbation, which can lead to preterm birth and stillbirth. In contrast, in the same review female neonates are suggested to adapt to poor intrauterine conditions by reducing their growth, therefore surviving in adverse environments with less oxygen than usual as they are smaller in size, consequently insinuating that female neonates are more resilient to stress as they are more adaptable [19]. Furthermore, another study by the same author proposed that sex-differences in fetal physiology may originate from discrepancies in fetal sex-steroid concentrations and its metabolites in cord blood, but did not find any significant differences in steroid concentrations between the sexes [20]. Additionally, it has been reported a higher salivary concentration of cortisol in mothers carrying male fetuses in gestational weeks 24–30, proposing that male fetal sex may affect maternal physiological distress by raising cortisol concentration, and possibly prompting postnatal outcomes [21].

In clinical practice, neonatal sex is not taken into consideration when assessing the risk of pregnancy or perinatal outcomes. Generally, maternal characteristics such as BMI, gestational diabetes, and preeclampsia among others are common conditions accounted for in pregnancy [22]. Our findings contribute to understanding sex-related differences in neonatal vulnerability and highlight the need for further investigation. Further studies and prospective validation are needed to determine the potential clinical relevance of these findings before implications for clinical practice and risk stratification can be established.

The primary strength of this study lies within the large sample size included in the database, comprising data from the Perinatal Revisions Syd covering most of the maternity units in the South of Sweden over a 20-year period, from the year 1995 to 2015, ensuring that even uncommon perinatal events in the population were represented. This may enhance the generalizability of our findings to comparable high-income settings. The strict validation criteria used to screen all umbilical cord blood pH values for source of origin (artery or vein), was another major strength of the study. This ensured the removal of erroneous values including possible single vessel samples [23] leading to increased robustness of our results. Inadvertently, this may also have introduced a degree of selection bias, as pH validation led to exclusion of almost a third of the original study population. However, this was a critical step in the data quality assurance process and is in-line with standardized routines established for UApH studies [24].

Limitations of the study include the lack of maternal comorbidity data, such as maternal hypertensive disorders, which precluded further adjustment of our analyses. Although multivariable analyses were performed to account for potential confounders, residual confounding by other variables cannot be excluded. For some outcomes, the adjusted effect estimates were modestly higher than the unadjusted estimates. This may reflect the relationships between the included covariates and the outcomes. However, given the observational design, residual confounding and model-related effects cannot be excluded. Furthermore, the multivariable analyses did not account for all potentially relevant obstetric confounding or mediating factors, including induction of labor and instrumental vaginal birth, which should be considered when interpreting the adjusted associations. BMI and smoking status also had a substantial proportion of missing data. As these variables were not included in the adjusted analyses, no imputation was performed, and the potential impact of the missing data on our findings cannot be excluded.

In addition, temporal changes in obstetric and neonatal care during the study period may have influenced the incidence and management of some neonatal outcomes. The available registry data did not provide detailed metadata regarding who entered individual variables, the precise timing of data entry, or measures of inter-center consistency. This is a limitation of the retrospective registry-based design. NICU admission was based on clinical judgment and local hospital guidelines, as uniform national admission criteria were not available during the study period, and the specific indication for admission could not be determined from the registry data. Outcome selection was limited by the neonatal variables systematically available in the registry. CPAP was routinely recorded, whereas reliable information on invasive mechanical ventilation was not available and could therefore not be assessed. Mortality data were not available in the dataset and could therefore not be assessed.

The registry also did not provide information on the underlying etiology of seizures, and therefore their cause in individual cases could not be determined. The predefined registry variable “CNS disease” did not distinguish between specific CNS diagnoses, which limits the clinical interpretation of this outcome. As follow-up in the register was limited to the neonatal period, CNS disorders diagnosed later in infancy or childhood were not captured. As multiple outcomes were analyzed, the findings, particularly those with modest effect sizes and p-values close to 0.05, should be interpreted with appropriate caution.

## Conclusions

In conclusion, this large, population-based cohort study demonstrates that male neonatal sex is associated with several adverse perinatal outcomes, including umbilical artery pH < 7.05, Apgar score < 7 at 5 min, the need for continuous positive airway pressure (CPAP), admission to the neonatal intensive care unit (NICU), hypoxic-ischemic encephalopathy (HIE), and central nervous system (CNS) disease. These findings add to the evidence of sex-related differences in neonatal outcomes. Further research is needed to establish whether these differences have implications for future clinical risk assessment and management.

## Data Availability

Data for this study were obtained from the Swedish Perinatal Revision South Register. Due to legal and ethical restrictions, the data cannot be made publicly available. Access may be granted upon reasonable request to the register authority and in line with Swedish data protection law.
